# Narrative review: clinical assessment of peripheral tissue perfusion in septic shock

**DOI:** 10.1186/s13613-019-0511-1

**Published:** 2019-03-13

**Authors:** Geoffroy Hariri, Jérémie Joffre, Guillaume Leblanc, Michael Bonsey, Jean-Remi Lavillegrand, Tomas Urbina, Bertrand Guidet, Eric Maury, Jan Bakker, Hafid Ait-Oufella

**Affiliations:** 1 Service de réanimation médicale, Assistance Publique-Hôpitaux de Paris (AP-HP), Hôpital Saint-Antoine, 184 rue du Faubourg Saint-Antoine, 75571 Paris Cedex 12, France; 2 Sorbonne Université, Université Pierre-et-Marie Curie-Paris 6, Paris, France; 3 Division of Critical Care Medicine, Department of Anesthesiology and Critical Care Medicine, Université Laval, Québec City, QC Canada; 4 Population Health and Optimal Health Practices Research Unit (Trauma – Emergency – Critical Care Medicine), Centre de recherche du CHU de Québec – Université Laval, Université Laval, Québec City, QC Canada; 5 Inserm U1136, Paris, 75012 France; 6 Department Intensive Care Adults, Erasmus MC University Medical Center, Rotterdam, The Netherlands; 7 Department of Pulmonology and Critical Care, Columbia University Medical Center, New York, USA; 8 Department of Pulmonology and Critical Care, New York University Medical Center – Bellevue Hospital, New York, USA; 9 Department of Intensive Care, Pontificia Universidad Católica de Chile, Santiago, Chile; 10 Inserm U970, Centre de Recherche Cardiovasculaire de Paris (PARCC), Paris, France

**Keywords:** Septic shock, Microcirculation, Capillary refill time, Temperatures gradient, Peripheral perfusion index, Mottling, Skin

## Abstract

Sepsis is one of the main reasons for intensive care unit admission and is responsible for high morbidity and mortality. The usual hemodynamic targets for resuscitation of patients with septic shock use macro-hemodynamic parameters (hearth rate, mean arterial pressure, central venous pressure). However, persistent alterations of microcirculatory blood flow despite restoration of macro-hemodynamic parameters can lead to organ failure. This dissociation between macro- and microcirculatory compartments brings a need to assess end organs tissue perfusion in patients with septic shock. Traditional markers of tissue perfusion may not be readily available (lactate) or may take time to assess (urine output). The skin, an easily accessible organ, allows clinicians to quickly evaluate the peripheral tissue perfusion with noninvasive bedside parameters such as the skin temperatures gradient, the capillary refill time, the extent of mottling and the peripheral perfusion index.

## Background

International guidelines emphasized that fast identification, assessment and treatment combining early antibiotic therapy, fluid administration and vasopressor infusion are crucial steps in the management of septic shock. However, despite early management, mortality of patients with septic shock remains high [1]. A possible explanation may be the persistent tissue hypoperfusion despite restoration of macro-hemodynamic parameters.

The usual hemodynamic targets for resuscitation of patients with septic shock use macro-hemodynamic parameters (heart rate, mean arterial pressure, central venous pressure). However, persistent alterations of microcirculatory blood flow despite restoration of macro-hemodynamic parameters can lead to organ failure. In a meta-analysis of 252 patients, De Backer et al. [2] showed that microcirculatory perfusion alterations predict mortality during serious infections, whereas mean arterial pressure or cardiac output did not. In critically ill patients, cardiac output optimization using increasing doses of dobutamine did not improve microvascular blood flow in the sublingual area [3, 4]. In another study, modulating mean arterial pressure by increasing norepinephrine dose had variable unpredictable effects on microcirculatory flow, which occasionally worsened [5, 6]. This dissociation between macro- and microcirculatory compartments, defined by Ince as «a loss of hemodynamic coherence» [7], brings a need to assess end organs tissue perfusion in patients with septic shock and to develop tools to analyze microcirculatory blood flow [8]. The direct identification of severe microcirculatory alterations remains difficult at bedside. Traditional markers of tissue perfusion may not be readily available (lactate) or may take time to assess (urine output). The skin, an easily accessible organ, allows clinicians to quickly evaluate the peripheral tissue perfusion with noninvasive bedside parameters such as the skin temperatures gradient, the capillary refill time, the extent of mottling and the peripheral perfusion index.

The aim of this review is to evaluate whether peripheral tissue perfusion assessment in septic patients could be helpful in evaluating organ failure severity and to screen patients at high risk of mortality. Finally, we analyze available data regarding implementation of peripheral perfusion evaluation in sepsis management.

### Skin as a tool for the evaluation of the microcirculation and tissue perfusion

**Fig. 1:**
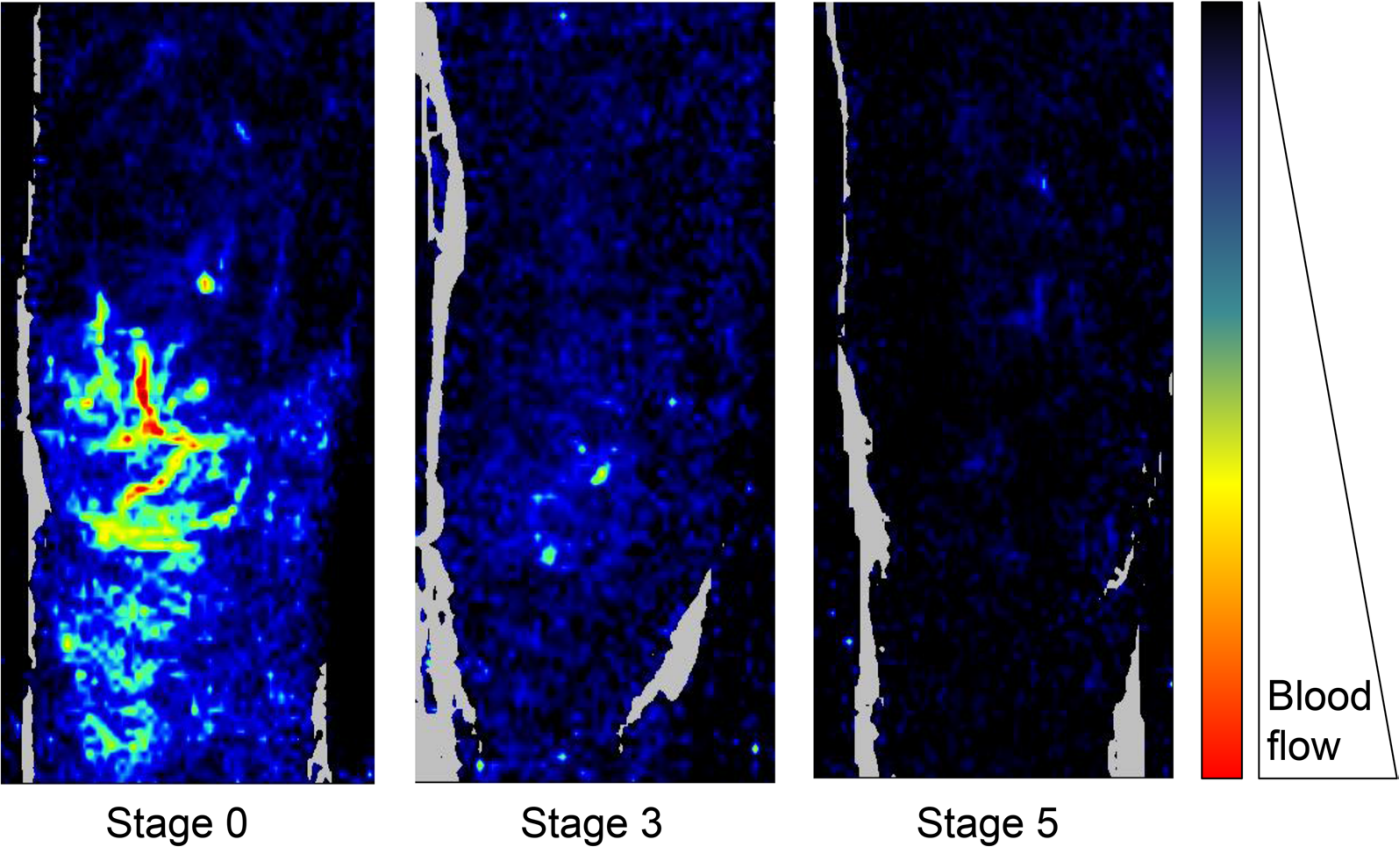
Examples of skin microvascular perfusion evaluation using laser Doppler imaging in the knee area according to the mottling score. Skin perfusion decreases when mottling score worsens. Adapted from [9]

**Fig. 2:**
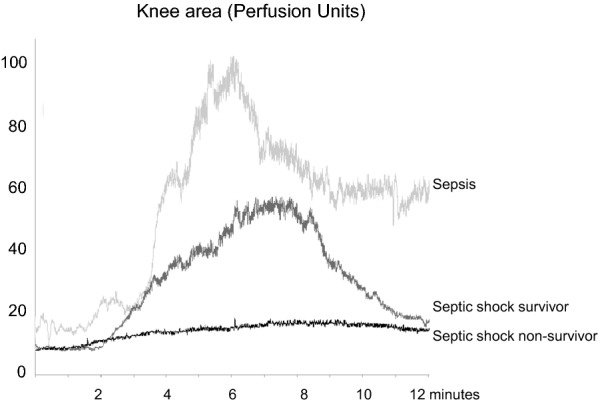
Examples of skin microcirculatory endothelial reactivity in the knee area in a patients with sepsis, in a patient with septic shock that was alive at day 14 and in a patient with septic shock that was ultimately dead at day 14. Skin microcirculatory blood flow was measured at baseline and after acetylcholine iontophoresis. Adapted from [11]

The skin provides important information in patients with septic shock. As a visible and easily accessible organ, the skin allows simple observation of local microcirculatory perfusion through skin temperature alterations (skin temperature gradient), perfusion (capillary refill time) and color (mottling). The pathophysiology of these clinical disorders has not been investigated in depth, but several authors assume that the main driven mechanism of reduced blood flow is local vasoconstriction mediated by sympathetic neuroactivation [8]. Additional mechanisms could participate to impair microvascular blood flow (Fig. 1) [9, 10] such as local endothelial dysfunction [11, 12] (Fig. 2), leukocyte adhesion, platelet activation and fibrin deposition [13]. These clinical, noninvasive, easy-to-use, parameters are attractive tools to follow microcirculatory perfusion in patients with acute circulatory failure [14, 15]. In 2014, several European experts recommended to integrate abnormal skin perfusion parameters in the definition and treatment of shock [16].

Subjective assessment of peripheral skin temperature may be a valuable tool in the evaluation of patients with septic shock. Eighty years ago, Ebert et al. [17] described the skin of septic shock patients as being «pale, often sweaty». Altemeier et al. [18] then noticed that a moist and cold skin was a factor of worse prognosis in patients with septic shock. Cold hands and feet, and abnormal skin color are the first clinical signs that developed in meningococcal disease in children [19]. In a cohort of 264 surgical ICU patients, patients with cold skin on extremities and knees had significantly lower central venous saturation and higher lactate level as compared to patients with normal skin temperature (4.7 ± 1.5 vs 2.2 ± 1.6 mmol/L, *p* < 0.05) [20]. In a prospective cohort study of 50 critically ill patients with circulatory dysfunction, including 26 patients with septic shock, Lima et al. [21] observed that patients with cold skin on the extremities had a higher rate of organ failure at 48 h after resuscitation as compared to patients with normal skin temperature.

However, skin temperature gradients may be more accurate in the evaluation of patients with septic shock. Several studies investigated quantitative temperature gradients in critically ill patients, particularly between peripheral and ambient temperatures [22], central and peripheral body temperatures [23] and finger and forearm skin temperatures [24]. Temperature gradients do not correlate with cardiac output [22, 25, 26] but are predictive of both organ failure severity and worse outcome. Joly et al. [22] measured toe-to-ambient temperature gradients 3 h after admission in a mixed population of critically ill patients, and non-survivors had a mean toe-ambient temperature gradient of 0.9 °C, whereas survivors had a gradient of 3.4 °C. Normalization of central-peripheral temperature gradients (< 7 °C) within the 6 first hours of resuscitation predicted correction of hyperlactatemia in septic shock patients [27]. In a recent study including 103 septic patients, Bourcier et al. [28] reported higher central-to-toe temperature gradients and lower toe-to-ambient temperature gradients in patients with septic shock, compared to patients with sepsis. Moreover, a rise in the toe-to-ambient temperature gradient was independently associated with decreased ICU mortality (OR 0.7 [0.5, 0.9] per °C, *p* < 0.001).

**Fig. 3:**
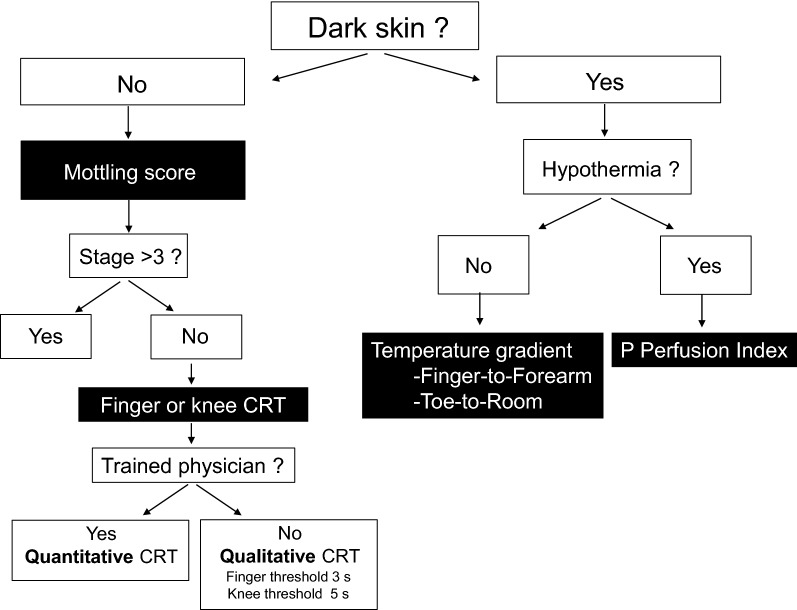
Proposed strategy to evaluate peripheral tissue perfusion using the skin. *CRT* capillary refill time, *P.Perfusion index* peripheral perfusion index

Finger-to-forearm skin and toe-to-ambient temperature gradients are more accurate tools that could be used in every patient without previous hypothermia, including patients with dark skin, providing quantitative information with good reproducibility (Table 1, Fig. 3).

**Table 1:** Summary of selected studies investigating clinical parameters of peripheral tissue in critically ill sepsis patients

Parameters	References	Patients’ number	% sepsis	% septic shock	Relation to organ failure severity	Relation to mortality	Changes following resuscitation
Peripheral temperature							
Subjective assessment: cold versus warm extremities	Kaplan et al. [20]	264	42	–	Cold extremities group had lower cardiac index, lower SvO2 and higher lactate levels	–	–
Toe-to-room temperature gradient	Joly et al. [22]	100	20			Temperature gradient lower in non-survivors	Temperature gradient increased in non-survivors following resuscitation but decreased in non-survivors
Toe-to-room temperature gradient	Bourcier et al. [28]	103	39	61	–	Lower in MOF death patients	Decreased in MOF death patients but increased in survivors
Capillary refill time (CRT)							
Finger-tip and knee CRT	Ait-Oufella et al. [35]	59	0	100	Correlated with SOFA score	Related to Day-14 mortality	CRT decreased during resuscitation which is associated with better outcome
Finger-tip CRT	Lara et al. [47]	95	100	–	–	–	Prolonged CRT following resuscitation is associated with higher organ failure severity and higher mortality
Finger-tip CRT	Hernandez et al. [46]	104	0	100	–	–	CRT is normalized within 6 h following resuscitation, whereas lactate normalization is longer
Mottling							
Mottling score after initial resuscitation	Ait-Oufella et al. [37]	60	0	100	Correlated with lactate, urinary output and SOFA score	Related to Day-14 mortality	Mottling score decreased following resuscitation which was associated with better outcome
	De Moura et al. [39]	97	0	100	–	Related to Day-28 mortality	–
	Preda et al. [41]	109	100	0	–	Related to Day-28 mortality	–
Mottling presence	Coudroy et al. [40]	791	–	–	–	Related to Day-28 mortality	Mottling persistence > 6 h was associated with higher mortality
Combined parameters							
Finger tip CRT + temperature gradient + peripheral perfusion index	Lima et al. [21]	50	–	42	Associated with lactate levels	–	Peripheral hypoperfusion associated with worsening SOFA score following resuscitation
CRT and central-to-toe temperature gradient	Hernandez et al. [27]	41	33	67	–	–	CRT is the first be normalized during resuscitation within 2 h

*CRT* capillary refill time, *MOF* multiorgan failure, *SOFA* sequential organ failure assessment

### Capillary refill time

The capillary refill time (CRT) measures the amount of time necessary for the skin to return to baseline color after applying a pressure on a soft tissue (generally finger tip). The CRT gives important information on skin perfusion and microcirculatory status but does not reflect cardiac output [25, 29]. Visual measurement of CRT associated with other clinical signs (tachycardia, mucosal dryness, etc.) helps to diagnose dehydration in children [30]. In acute pathologies, such as gastro-intestinal infections or malaria [31], CRT represents an attractive and easy-to-use tool for clinicians in the initial screening of severely ill patients [32]. Inter-rater variability of CRT was weak in non-trained physicians [33], but is better in centers expert in tissue perfusion evaluation [34], especially in the knee area [35]. Standardization of finger-tip pressure (i.e., How long? How strong the applied pressure?) might improve CRT reproducibility. Ait-Oufella et al. [35] obtained good inter-rater concordance by “applying a firm pressure for 15 s. The pressure applied was just enough to remove the blood at the finger tip of the physician’s nail illustrated by appearance of a thin white distal crescent (blanching) under the nail.”

Capillary refill time measurement correlates with the pulsatility index, a surrogate ultrasound-derived parameter that reflects vascular tone of visceral organs in septic shock patients [36]. CRT is an interesting tool to assess the severity of an acute illness. In the intensive care unit, Lima et al. [21] reported an association between a prolonged CRT (> 4.5 s on the index finger) and hyperlactatemia and a higher SOFA score. In septic shock patients, a prolonged CRT 6 h after resuscitation has been shown to be predictive of 14-day mortality, with an Area Under Curve (AUC) of 84% for a measure on the index finger, and 90% for a measure on the knee. A 2.4-second threshold value on the index finger predicted mortality with an 82% sensitivity (95% CI [60–95]) and a 73% specificity (95% CI [56–86]). On the knee, a threshold value of 4.9 s predicted 14-day mortality with an 82% sensitivity (95% CI [60–95]) and an 84% specificity (95% CI [68–94]) [35].

Overall, when used as a qualitative variable (prolonged or not), CRT is a reliable triage tool to identify critically ill patients at risk of negative outcome. Quantitative measurement of CRT should be mainly used by trained physicians in patients with non-dark skin (Table 1, Fig. 3).

### Mottling

Mottling, a characteristic discoloration of the skin following reduced skin blood flow [9], is taught as a marker of shock, but its clinical relevance has been poorly investigated until recent years. A significant relationship between mottling extension and visceral organ vascular tone has been reported suggesting that mottling could reflect gut, liver spleen and kidney hypoperfusion [36].

**Fig. 4:**
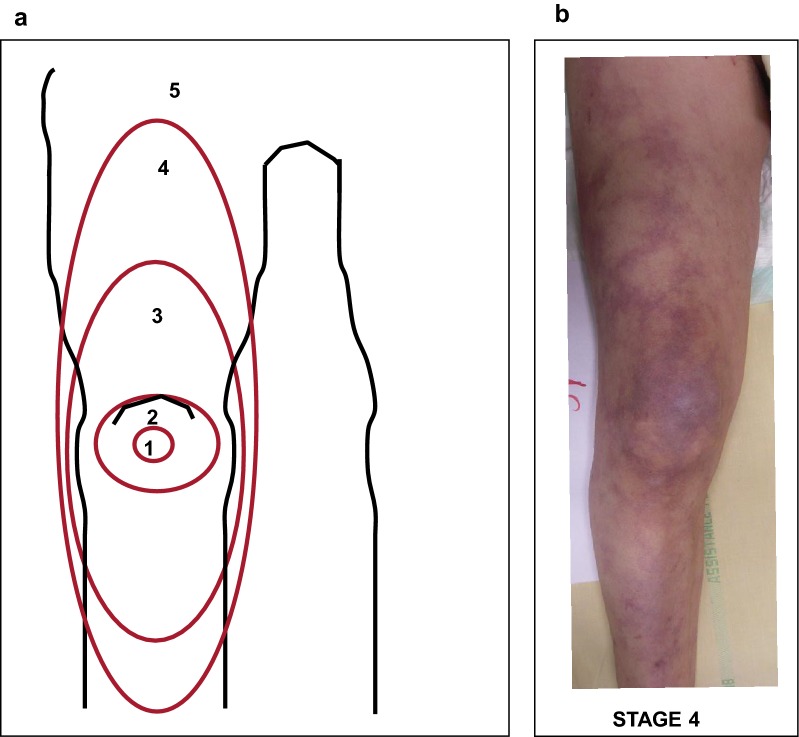
**a** The mottling score, ranging from 0 to 5, is based on skin mottling area extension on legs. Score 0 represents no mottling, score 1 represents small mottling area (coin size) localized to the center of the knee, score 2 represents mottling area not exceeding the superior edge of the knee cap, score 3 represents mottling area not exceeding the middle thigh, score 4 represents mottling area not exceeding the fold of the groin and score 5 otherwise. **b** Example of mottling score 5. Adapted from [37]

To assess the predictive value of mottling in critically ill patients with severe infections, a semi-quantitative clinical score for mottling (ranging from 0 to 5), based on the extension of these purple patches from the patella toward the periphery, has been developed and validated with an excellent inter-observer reproducibility [37] (Kappa 0.87% (CI 95% [0.72–0.97]) (Fig. 4). Mottling score reliably reflects organ failure severity in patients with sepsis or septic shock and helps to identify critically ill patients with worse outcome. In a study including septic shock patients, the mottling score at 6 h after resuscitation was predictive of death at day 14 (odds radio [OR] 16, CI 95% 4–81, for stages 2–3; vs 74, CI 95% 11–1568, for stages 4–5). Mortality occurred within 12–24 h for stages 4–5, within 24–72 h for stages 2–3 and later than 72 h for the rare deaths for stages 0–1 (Kaplan–Meier charts, *p* < 0.0001). In the same study, cardiac output and blood pressure were not associated with mortality at day 14, confirming the disparity between microcirculatory and macrocirculatory parameters [37]. These results were confirmed in cirrhotic patients with septic shock [38]. In addition, in mottling groups ≤ 3, knee CRT improved patient discrimination according to their outcome, with non-survivors presenting a significantly higher knee CRT [35]. Another South American study confirmed these results in septic shock patients. Mortality rate at day 28 was 100% when the mottling score was higher or equal to stage 4, 77% for stages 2 and 3, and 45% for stages 1 or lower [39]. Prognostic value of mottling was also reported in unselected ICU patients: Persistent (> 6 h) mottling extending over the knee (> stage 2) was an independent risk factor for mortality (OR 3.29, 95% CI 2.08–5.19; *p* < 0.0001) [40]. Finally, Preda et al. [41] found the good predictive value of the mottling score for mortality at day 28 in patients with sepsis not receiving vasopressors.

In summary, mottling score is a reliable semi-quantitative tool that reflects organ failure severity in non-selected septic patients with or without vasopressors and is helpful to identify critically ill patients with pejorative outcome and also to monitor changes during resuscitation. In patients with mottling score ranging from 0 to 3, knee CRT measurement could be associated with improving risk stratification (Table 1, Fig. 3).

### Peripheral perfusion index

Peripheral perfusion index is defined as the difference between the pulsatile and non-pulsatile portion of pulse wave, measured by plethysmography. Peripheral perfusion index (PPI) gives information on peripheral vascular tonus by the pulsatility, decreasing in vasoconstriction and raising in vasodilation [42]. Peripheral perfusion index is an early predictor of central hypovolemia [43]. In a prospective observational study in an emergency department, PPI was not significantly different between patients admitted to the hospital and patients discharged from the emergency department suggesting that it could not be used as a triage tool [44]. However, in critically ill patients, PPI is significantly lower in patients with a peripheral perfusion alteration (0.7 vs 2.3, *p* < 0.01) [21]. He et al. [45] showed that the PPI is altered in septic shock patients, as compared to control subjects in postoperative scheduled surgery. Moreover, in the same study, the PPI was significantly lower in non-survivors. With a 0.20 cutoff value, PPI was predictive of ICU mortality with an AUC of 84% (69–96), a sensitivity of 65% and a specificity of 92%.

## Discussion

### Abnormal skin perfusion evaluation and resuscitation

Despite some differences between micro and macrovascular compartments, it would be over-simplifying and possibly wrong to completely separate these two vascular compartments. In the study by Ait-Oufella et al. [37] focusing on mottling, global hemodynamic improvement within the first hours following resuscitation, based on blood volume optimization and catecholamine use, was associated with mottling improvement. Patients whose mottling score improved through the first 6-hour resuscitation had a good prognosis, whereas those whose score was stable or even worsened had a poor prognosis (14-day mortality: 23% vs 88%, *p* < 0.001). Finger-tip CRT is also quickly normalized in septic shock patients within 2–6 h after resuscitation, whereas hyperlactatemia requires longer time to recover [27, 46]. Interestingly, patients in whom CRT did not recover after fluid infusion had pejorative outcome [47]. Altogether, these studies suggest that peripheral tissue perfusion could be used as triage tool at the early steps of sepsis management at admission and after fluid infusion. The ongoing ANDROMEDA-SHOCK trial aims to compare two resuscitation strategies during the first hours of sepsis treatment on 28-day mortality, one based on CRT measurement and the other on arterial lactate clearance [48]. During ICU stay, evaluation of peripheral perfusion could also be helpful. A «proof-of-concept» study has been done comparing a volume expansion strategy based on peripheral perfusion, clinical parameter assessment, to a classical strategy based on mean arterial pressure, central venous pressure and cardiac index. Peripheral perfusion was assessed through CRT, index-forearm temperature gradient, peripheral perfusion index, and StO2. The resuscitation strategy based on clinical tissue perfusion assessment led to a reduction in fluid therapy volume in the first 72 h (7565 ± 982 mL vs. 10,028 ± 941 mL, *p* = 0.08) and to a reduction in hospital length of stay (16 [5–28] vs. 43 [8–45] days, *p* < 0.05) [49]. A task force of six international experts with extensive bedside experience recently proposed to integrate peripheral tissue perfusion tools in risk stratification and management of septic patients in resource-limited intensive care units, especially CRT, mottling score and temperature gradients [50].

As bedside evaluation of tissue perfusion using the skin improves risk stratification in patients with sepsis, there is a possibility that it could be used as a tool to guide resuscitation. Lavillegrand et al. [51] reported that a mild arterial hypotension (MAP between 55 and 65 mmHg) could be safely tolerated in patients without any sign of hypoperfusion. Such «personalized» management requires close monitoring (in an ICU) but may decrease the use of invasive devices and vasopressors, both having potential side effects. Conversely, patients with markers of tissue hypoperfusion require rapid ICU transfer, and also, we hypothesized that they should be good candidate for therapeutic approaches targeting microcirculation for resuscitation in the future. For example, nitroglycerin infusion had no beneficial effect in unselected sepsis patients [52] but improved peripheral perfusion in selected patients with prolonged CRT and/or increased finger-tip-to-forearm skin gradient temperatures [53]. Ilomedin has been also recently proposed as a rescue therapy in sepsis shock with refractory tissue hypoperfusion [54] and will be tested soon in a prospective randomized multicenter trial (I-MICRO NCT03788837). In the future, it is important to evaluate whether drugs targeting the microcirculation could improve outcome of selected patients with persistent peripheral hypoperfusion despite initial resuscitation [55]. The first results of ANDROMEDA-SHOCK, an international multicenter trial recently completed, support that a tissue perfusion-guided resuscitation is beneficial [48, 56]. Indeed, Hernandez et al. [56] showed in septic shock adults that an early peripheral perfusion-targeted resuscitation, aiming at normalizing capillary refill time, was associated with less organ dysfunction at day 3 and a trend toward reduced 28-day mortality when compared to a lactate-level-targeted therapeutic strategy.

### Limitations

In this review, almost all data were obtained in small-sized monocenter observational studies and were performed by experts in tissue perfusion evaluation, suggesting potential biases. In addition, no published multicenter randomized trial is available showing that the implementation of bedside tissue perfusion assessment improves septic patients management and *in fine* outcome. This narrative review did not provide strong recommendation regarding the use of tissue perfusion parameters in septic patients according to GRADE methodology but only proposed how and when to implement them.

## Conclusion

In patients with septic shock, tissue microvascular hypoperfusion can be evaluated at bedside using indicators of skin perfusion. After initial resuscitation, these parameters are helpful in identifying patients with severe organ failure and at high risk of mortality. However, there is a need in the future to investigate these bedside tissue microvascular perfusion parameters as management targets for resuscitation in septic shock patients.

## Abbreviations

CI: confidence interval
CRT: capillary refill time
ICU: intensive care unit
MAP: mean arterial pressure
NIRS: near-infrared spectroscopy
NO: nitric oxide
OR: odds ratio
PPI: peripheral perfusion index
SOFA: sequential organ failure assessment
SAPS II: Simplified Acute Physiologic Score II
ROC: receiver operating characteristics
